# Mapping quantitative trait loci associated with common bunt resistance in a spring wheat (*Triticum aestivum* L.) variety Lillian

**DOI:** 10.1007/s00122-019-03403-3

**Published:** 2019-08-13

**Authors:** Firdissa E. Bokore, Richard D. Cuthbert, Ron E. Knox, Arti Singh, Heather L. Campbell, Curtis J. Pozniak, Amidou N’Diaye, Andrew G. Sharpe, Yuefeng Ruan

**Affiliations:** 1 Swift Current Research and Development Center, Agriculture and Agri-Food Canada, Swift Current, SK S9H 3X2 Canada; 2 Department of Agronomy, Iowa State University, Ames, IA USA; 3 Department of Plant Sciences, University of Saskatchewan, Saskatoon, SK S7N 5A8 Canada; 4 National Research Council of Canada, 110 Gymnasium Place, Saskatoon, SK S7N 0W9 Canada

## Abstract

**Key message:**

Based on their consistency over environments, two QTL identified in Lillian on chromosomes 5A and 7A could be useful targets for marker assisted breeding of common bunt resistance.

**Abstract:**

Common bunt of wheat (*Triticum aestivum* L.) caused by *Tilletia tritici* and *T. laevis* is an economically important disease because of losses in grain yield and reduced grain quality. Resistance can be quantitative, under the control of multiple small effect genes. The Canada Western Red Spring wheat variety Lillian is moderately resistant to common bunt races found on the Canadian prairies. This study was conducted to identify and map quantitative trait loci (QTL) conferring resistance against common bunt in Lillian. A doubled haploid population comprising 280 lines was developed from *F*_1_ plants of the cross of Lillian by Vesper. The lines were inoculated at seeding with the two races L16 (*T. laevis*) and T19 (*T. tritici*), grown in field near Swift Current, SK, in 2014, 2015 and 2016 and assessed for disease incidence. The lines were genotyped with the 90 K iSelect SNP genotyping assay, and a high-density genetic map was constructed. Quantitative trait locus analysis was performed with MapQTL.6^®^ software. Two relatively stable common bunt resistance QTL, detected in two of the 3 years, were identified on chromosomes 5A and 7A from Lillian. In addition, three less stable QTL, appearing in one out of 3 years, were identified: one was contributed by Lillian on chromosome 3D and two were contributed by Vesper on chromosomes 1D and 2A. Epistatic interaction was identified for the bunt incidence between 3D and 7A resulting in greater bunt resistance. Future bunt resistance breeding will benefit from combining these QTL through gene pyramiding.

## Introduction

Common bunt (syn. stinking smut) caused by *Tilletia tritici* (Bjerk.) Wint. and *T. laevis Kühn* is a serious disease, reducing yield and quality in wheat (*Triticum aestivum* L.) (Gaudet and Menzies 2012; Goates 1996; Hoffmann 1982). The disease is initiated by teliospores on the seed or in the soil germinating and infecting the developing seedling. The fungus progresses systemically in the plant, eventually replacing kernels with bunt balls containing masses of spores (Gaudet et al. 1993). Yield loss due to common bunt is approximately equivalent to the percentage of infected tillers (Menzies et al. 2006). Loss in grain quality occurs in grain contaminated with bunt balls at levels as low as 0.05% by weight. The disease remains a serious problem to wheat production worldwide despite the extensive amount of research conducted (Gaudet and Menzies 2012; Goates 1996). Common bunt is most notably a problem in organic wheat production because traditional seed treatment fungicides are not permitted (Gaudet and Menzies 2012).

In addition to wheat, which is the primary host of common bunt, other cereals such as rye and barley and several grasses can serve as hosts (Gaudet and Menzies 2012). Host adaptation of *Tilletia* species may exist within wheat and other crops. For example, Mamluk (1998) reported that *T. laevis* predominates in bread wheat, whereas *T. tritici* infects both bread and durum nonpreferentially. Apart from host resistance, seed treatment fungicides and cultural practices can reduce common bunt significantly (Gaudet and Menzies 2012; Knox et al. 2013). However, fungicides are expensive, have toxicity issues, present an environmental hazard and have availability and distribution challenges (Goates 1996). The use of resistant varieties offers the best solution for the control of bunt. Genetic resistance to common bunt is relatively easy to select because high infection levels are possible in inoculated field trials (Gaudet and Menzies 2012).

Most Canadian hard red spring wheat varieties grown prior to 1940 were susceptible to common bunt; however, the majority of varieties developed since that time possess intermediate to high levels of resistance (Gaudet et al. 1993; Gaudet and Puchalski 1989a). An understanding of the genetic control in contemporary Canadian wheat varieties carrying common bunt resistance genes is developing (Fofana et al. 2008; Gaudet et al. 1993, 2007; He and Hughes 2003; Knox et al. 2013; Singh et al. 2016; Wang et al. 2009).

Over 15 common bunt major resistance genes *Bt1* through *Bt15* and *Btp* have been designated in wheat (Goates 1996, 2012). Among these, *Bt10* is effective against all known naturally occurring common bunt races globally and is an important resistance source for new wheat varieties (Chen et al. 2016; Demeke et al. 1996; Gaudet et al. 2007). Several of the designated common bunt resistance genes have known chromosomal location. *Bt10* is genetically mapped to the terminal end of chromosome 6DS (Menzies et al. 2006) and *Bt9* mapped as a distinct factor on the distal end of chromosome 6DL (Steffan et al. 2017). Wad and Metzger (1970) reported that *Bt1* is located on chromosome 2B, and *Bt4* and *Bt6* are located on chromosome 1B linked with the gene that regulates red glume color.

Quantitatively inherited resistance to common bunt also exists (Fofana et al. 2008). Quantitative trait loci (QTL) associated with common bunt resistance were reported on several wheat chromosomes: two QTL were identified on chromosome 1B and a third on chromosome 7A in AC Domain (Fofana et al. 2008), 1BS in Blizzard (Wang et al. 2009), 7B in McKenzie (Knox et al. 2013), 2B and 6A in Kenyon (McCartney et al. 2013), 1B, 4B, 4D, 5B and 7D in Carberry (Singh et al. 2016) and 1A, 2B and 7D in Idaho 444 (Chen et al. 2016). Dumalasová et al. (2012) reported a strong bunt QTL on chromosome 1B with other smaller effect QTL expressed on 5B, 7A and 7D in a European winter wheat variety Trintella.

Mapping genes for resistance to common bunt in wheat is valuable for gene pyramiding and deployment in future varieties. Molecular markers enable pyramiding resistance genes of interest into a single germplasm minimizing the need for greenhouse or field evaluation, thereby greatly simplifying the screening and selection process (Gaudet and Menzies 2012). Gene pyramiding could increase the longevity and effectiveness of resistance. The objective of this study was to identify and map quantitative trait loci (QTL) conferring resistance against common bunt in the wheat cultivar Lillian.

## Materials and methods

### Plant materials

A population of 280 DH lines developed from *F*_1_ plants of the cross Vesper/Lillian at the Swift Current Research and Development Centre with the maize by wheat system (Humphreys and Knox 2015) was used for this study. At the time of its commercial release, Lillian was moderately resistant to common bunt (DePauw et al. 2005), while Vesper was moderately susceptible (Thomas et al. 2013).

Similar to the procedure described by Knox et al. (2013), the DH lines, parents and bunt checks were evaluated for common bunt reaction in an experiment planted in a disease nursery established near Swift Current, SK (50°15′38″N 107°44′28″W), in each of the years 2014, 2015 and 2016. The experiments were planted in moist soil of a field that was summer fallow the previous growing season early in the spring to obtain cool soil conditions that favor common bunt development. Planting was performed on April 25, 2014 with a 5-cm soil depth mean temperature of 6 °C, on April 17, 2015 with a 5-cm soil depth temperature of 6 °C and on April 20, 2016 with a 5-cm soil depth temperature of 9 °C. Prior to planting, seeds of the lines, parents and checks were inoculated with spores of two common bunt races, *L16* (*T. laevis*) and *T19* (*T. tritici*) (Goates 2012; Singh et al. 2016). The *L16* and *T19* races together represent the entire bunt virulence spectrum in Canada (Gaudet and Puchalski 1989b; Singh et al. 2016).

In each of the 3 years, the DH lines, parents and bunt checks were planted in a completely randomized design of 3-m-long rows seeded at a rate of 200 seeds per row spaced 0.46 m apart (135 seeds m^−2^). The bunt checks Biggar (DePauw et al. 1991), Katepwa (Campbell and Czarnecki 1987), Neepawa (Campbell 1970), AC Barrie (McCaig et al. 1996), AC Cadillac (DePauw et al. 1998) and AC Elsa (Clarke et al. 1997) were repeated six times in each nursery with the exception of Neepawa that was not included in the 2016 nursery. The parents appeared once in the 2014 and 2015 nurseries; in 2016, each parent was repeated six times. Biggar was used as a bunt-susceptible check (Singh et al. 2016). The other checks have varying phenotypic expression, ranging from moderately susceptible to resistant. Disease incidence was recorded as a percentage of bunt-infected spikes over total number of spikes (percent bunt incidence = number of infected spikes/total number of spikes of a genotype * 100) in the row based on a visual assessment when plants reached the hard dough stage. Disease rating was repeated within each season to optimize assessment of both early and late developing lines. Where there were multiple checks or parental lines in a test, the mean of that line was used in data analysis. PROC ANOVA with a Duncan multiple range test was performed to compare bunt incidence between lines and checks using SAS (SAS institute, Cary, NC) with all data, using year as replicate.

### Genotyping and QTL mapping

The DNA of parents and Vesper/Lillian population lines was extracted from young leaves using Daisy 96 Plant Kits (QIAGEN Science, Maryland, USA) and genotyped with the 90 K iSelect SNP genotyping assay (Illumina Inc., San Diego, CA). Genotypic data were curated to remove monomorphic and highly distorted markers according to an expected 1:1 ratio for the population using the Chi-square test. A linkage map consisting of 7841 single-nucleotide polymorphism (SNP) markers spanning 3679.5 cM was built for the population using the two-step mapping strategy previously described (Fowler et al. 2016; Perez-Lara et al. 2016). The A genome had 3144 markers spanning 1643.1 cM length, the B genome had 3596 markers covering 1398.8 cM and the D genome had 1099 markers over 637.6 cM. All chromosomes were represented in the map. Simple interval mapping (SIM) followed by multiple QTL mapping (MQM) analysis was performed using a set of 1975 nonoverlapping SNP markers using MapQTL.6 ^®^ (Van Ooijen 2009). Where markers overlapped at specific map positions, the marker with results across the most lines of the population was retained and the redundant markers were removed.

Epistasis interactions between the QTL for bunt resistance were analyzed using QTL-Network v.2 as previously described, and critical *F* values were determined with 1000 permutations (Singh et al. 2014; Yang et al. 2007). The “two-dimensional (2D) genome scan” option was used to map epistatic QTL with or without single-locus effects. To estimate effects of the additive × additive (A × A) interaction in a doubled haploid population, the “map epistasis” option was used. A *t* test analysis comparing 3 years’ least square means of bunt incidence data for 32 groups of lines carrying varying numbers of common bunt resistance QTL was run using the LSMEANS statement with the PDIFF option of the PROC GLM in SAS software (SAS Institute, Cary, NC). Mean comparisons were made using the hypothesis [Pr > |*t*| for H0: LSMean (column group *i*) = LSMean (row group *j*)].

## Results

### Bunt incidence

**Fig. 1:**
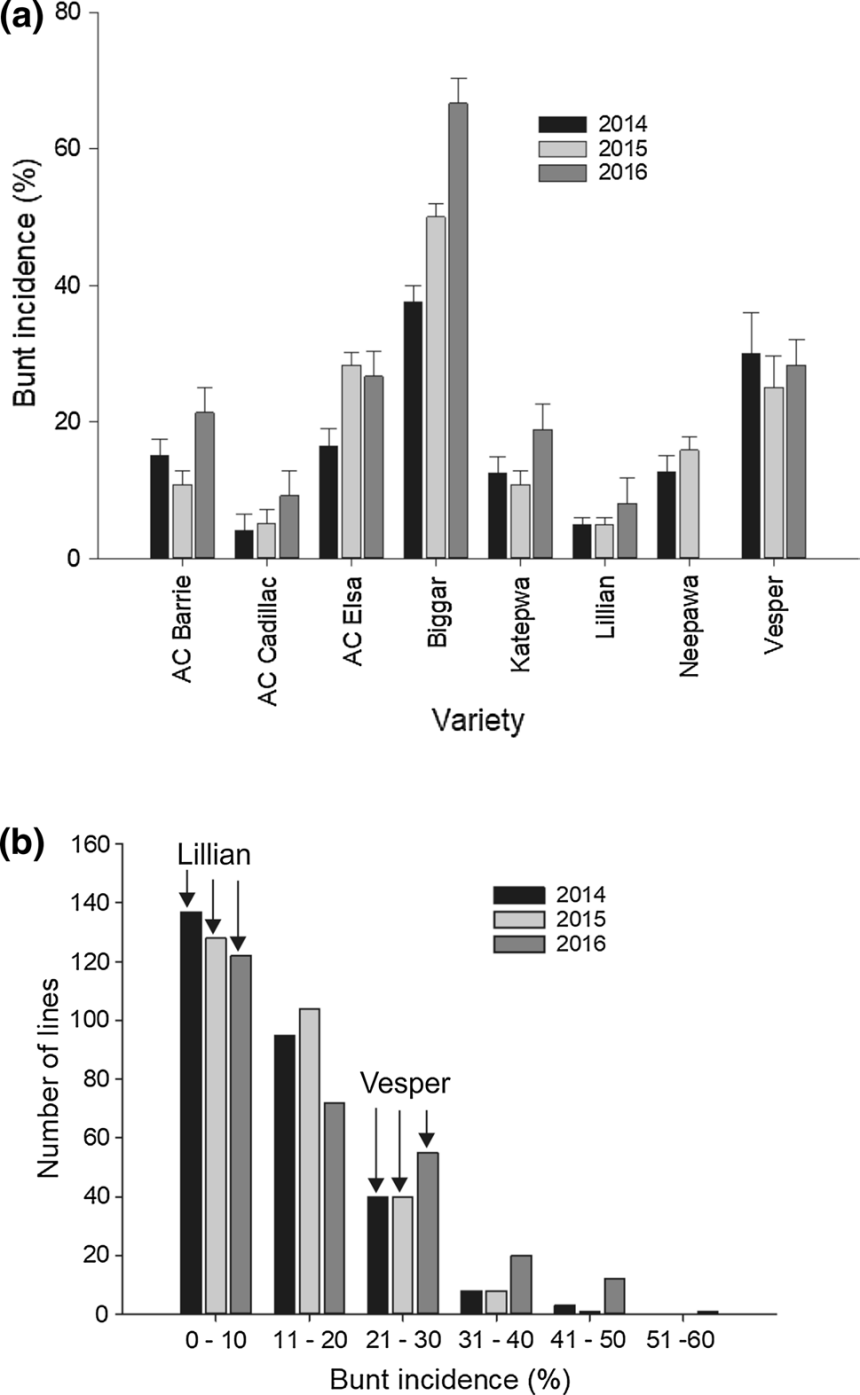
Swift Current, SK field nursery common bunt percent incidence in 2014, 2015 and 2016 of: **a** parents and check varieties and **b** plot of frequency distribution of the doubled haploid lines (*n* = 280) of the “Vesper”*/*“Lillian” cross along with the incidence of parental lines Lillian and Vesper designated by arrows for each year of testing

When comparing results of the phenotypic data of the parents and checks, the moderately resistant parent Lillian ($\bar{x}$ = 6.0%) and the resistant check AC Cadillac ($\bar{x}$ = 6.1%) had the lowest disease incidence, while the susceptible check Biggar displayed the highest disease incidence ($\bar{x}$ = 51.4%) over all 3 years (Fig. 1a; Supplementary Table 1). Check varieties with intermediate resistance Katepwa ($\bar{x}$ = 14.0%), Neepawa ($\bar{x}$ = 14.3%—2 years data) and AC Barrie ($\bar{x}$ = 15.7%) showed lower disease incidence than moderately susceptible Vesper ($\bar{x}$ = 27.8%) and AC Elsa ($\bar{x}$ = 23.8%). The mean disease incidence of Lillian was not statistically different from other checks except Vesper and Biggar. The distribution of the population lines for disease response was continuous with a majority of the lines showing resistance over the 3 years (Fig. 1b). No line demonstrated complete resistance. Four of the 280 DH lines (AJ091, AC037, AN089 and AH055) consistently had lower disease incidence than Lillian in each of the 3 years; this difference was statistically nonsignificant. The susceptible check Biggar was more susceptible than the most susceptible population line in 2016. Biggar also showed a similar reaction to the most susceptible line in 2015 and was relatively less susceptible in 2014. Biggar had significantly higher disease incidence than the parents, checks and all population lines except AC066, AN021, AC085 and AC005. These four lines were also not significantly different from Vesper. Bunt incidence of the DH lines ranged from 0 to 45% in 2014, 1 to 50% in 2015 and 3 to 60% in 2016 (Fig. 1b; Supplementary Table 1). The mean incidence of the population was 14.3% in 2014, 14.2% in 2015 and 18.0% in 2016.

The rainfall during the plant growth period (April–July) at the experimental site was 222 mm for 2014, 141 mm for 2015 and 372 mm for 2016 with the monthly distributions indicated in Supplementary Fig. 1. The disease score of the lines was generally higher for the wetter season than drier years.

### QTL mapping

**Fig. 2:**
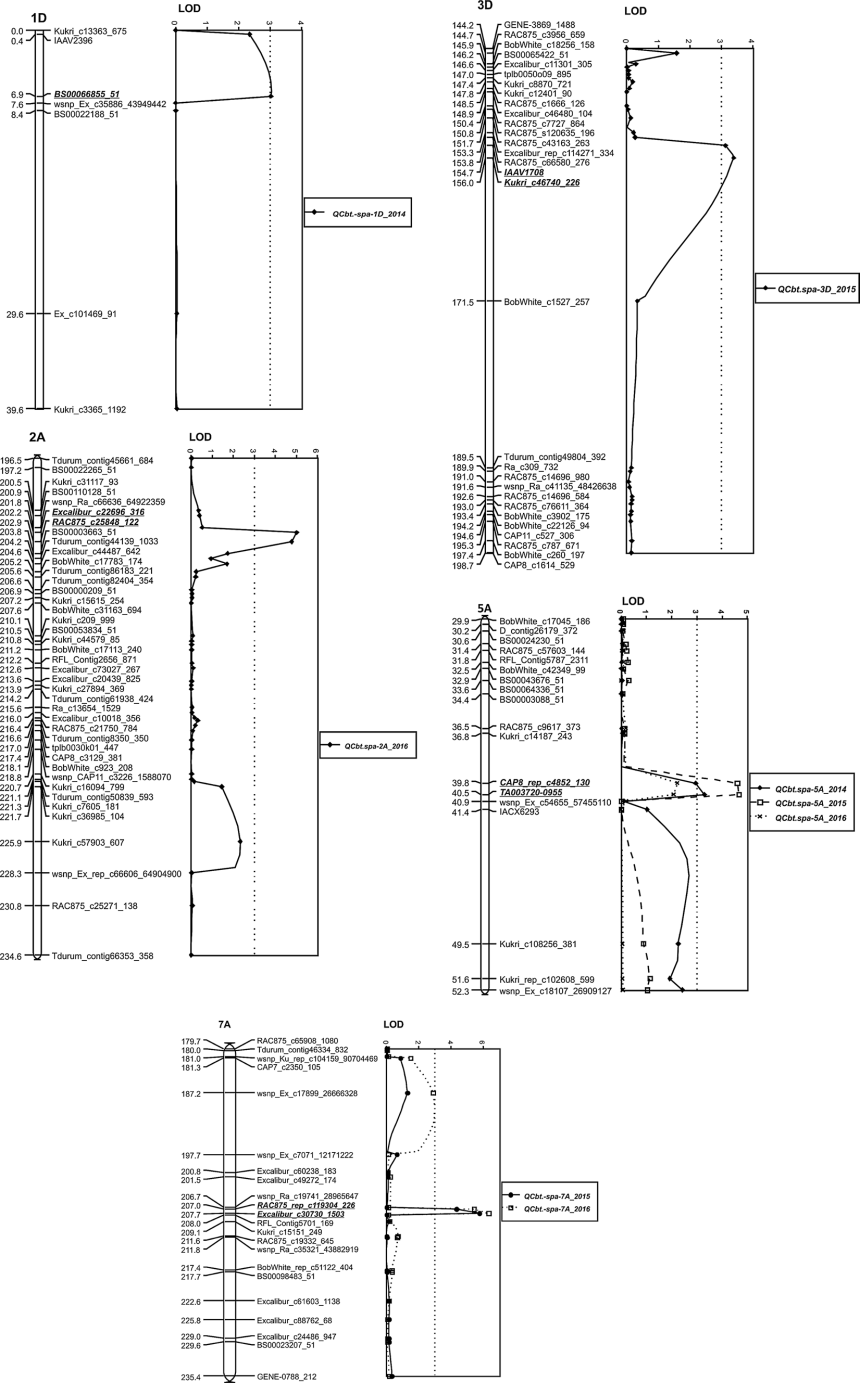
Linkage maps displaying five QTL associated with common bunt resistance contributed by Lillian on chromosomes 3D, 5A and 7A, and Vesper on 1D and 2A. (Based on population size, markers less than 0.4 cM apart are not reliable.) LOD values were generated using Multiple QTL Mapping (MQM) analysis. The column on the left is the map distance in cM corresponding to the 90 K wheat iSelect markers (Illumina Inc., San Diego, CA) in the center on the Vesper/Lillian population genetic map and LOD score on the right

The QTL mapping revealed five loci associated with common bunt resistance on chromosomes 1D, 2A, 3D, 5A and 7A (Table 1, Fig. 2). The location of the SNP markers that detected the common bunt resistance QTL in the International Wheat Genome Sequencing Consortium (IWGSC) RefSeq v1.0 wheat genome assembly is given in Supplementary Table 2. The QTL located on chromosomes 3D (*QCbt.spa*-*3D*), 5A (*QCbt.spa*-*5A*) and 7A (*QCbt.spa*-*7A*) were contributed by the moderately resistant Lillian. The remaining two QTL located on chromosomes 1D (*QCbt.spa*-*1D*) and 2A (*QCbt.spa*-*2A*) were contributed by the moderately susceptible Vesper. Both *QCbt.spa*-*5A* and *QCbt.spa*-*7A* QTL were statistically significant in two environments, *QCbt.spa*-*5A* being revealed in 2014 and 2015 and *QCbt.spa*-*7A* in 2015 and 2016. In 2016, the LOD score of *QCbt.spa*-*5A* was elevated but not significant, being 2.2 as compared with the genome-wide threshold of 3.4. Quantitative trait loci that appeared in one out of three test environments were on 1D in 2014, 3D in 2015 and 2A in 2016.

**Table 1:** Quantitative trait loci controlling common bunt identified in the Vesper/Lillian doubled haploid population, source of resistance alleles, peak SNP markers and associated phenotypic variation explained by each QTL at Swift Current, SK, in 2014, 2015 and 2016

Chromosome	Marker or markers at QTL peak	Position (cM)	LOD^1^	Lillian^a^ % bunt	Vesper^b^ % bunt	PVE (%)	Additive effect	Source of resistance allele
Swift Current 2014								
*1D*	*BS00066855_51*	6.9	3.0	16.6	12.4	4.8	2.1	Vesper
*5A*	*CAP8_rep_c4852_130*–*TA003720*-*0955*	39.8–40.5	3.3	12.2	16.6	5.3	− 2.2	Lillian
Swift Current *2015*								
*3D*	*IAAV1708*–*Kukri_c46740_226*	154.7–156.0	3.9	12.3	16.3	5.4	− 2.1	Lillian
*5A*	*CAP8_rep_c4852_130*–*TA003720*-*0955*	39.8–40.5	4.7	11.8	16.7	7.4	− 2.5	Lillian
*7A*	*RAC875_rep_c119304_226*–*Excalibur_c30730_1503*	207.0–207.7	5.8	10.3	16.9	9.1	− 3.3	Lillian
Swift Current *2016*								
*2A*	*Excalibur_c22696_316*–*RAC875_c25848_122*	202.2–202.9	5.0	22.6	13.2	7.9	5.1	Vesper
*5A*	*CAP8_rep_c4852_130*–*TA003720*-*0955*	39.8–40.5	2.2	15.7	20.7	3.6	− 2.3	Lillian
*7A*	*RAC875_rep_c119304_226*–*Excalibur_c30730_1503*	207.0–207.7	6.3	12.3	21.5	9.9	− 4.6	Lillian

*PVE* phenotypic variation explained

^a, b^Mean bunt incidence of lines of the population pooled by molecular variant of the stated parent

^1^Significant at 5% threshold level with the exception being the 5A QTL in 2016 which appeared but was not significant

Despite differences in stability over the three environments, the phenotypic variation explained by each of the five QTL was similar (Table 1). The highest explained incidence variation was 9.1% in 2015 for QTL *QCbt.spa*-*7A* associated with marker *RAC875_rep_c119304_226* and 9.9% in 2016 for *Excalibur_c30730_1503*. The lowest was 4.8% of the phenotypic variation explained for QTL *QCbt.spa*-*1D* associated with *BS00066855_51*.

**Fig. 3:**
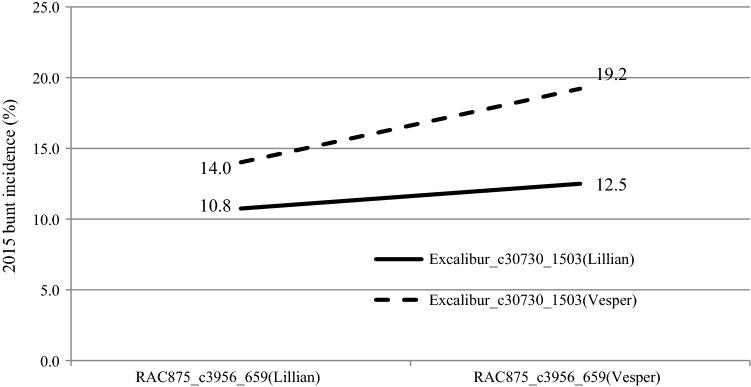
Epistatic interaction of common bunt incidence (%) between SNP markers *RAC875_c3956_659* on chromosome 3D and *Excalibur_c30730_1503* on chromosome 7A in 2015; both QTL on 3D and 7A were derived from Lillian

Three of the main effect QTL detected by MapQTL on chromosomes 3D, 5A and 7A were similarly detected by QTL-Network, whereas the two other QTL on 1D and 2A identified by MapQTL were not revealed. Significant additive × additive epistatic interactions for percent bunt incidence occurred between *QCbt.spa*-*3D *× *QCbt.spa*-*7A* in 2015 and the mean of 3 years (Table 2). Figure 3 displays the additive × additive epistasis interaction between 3D and 7A and the effect on bunt incidence in 2015. Without the 3D and 7A resistance allele, the bunt incidence was 19.2%. The epistatic interaction between the 3D Lillian allele at SNP marker *RAC875_c3956_659* and the 7A Lillian allele at SNP marker *Excalibur_c30730_1503* resulted in 10.8% bunt incidence as compared with individual effects of 12.5% bunt incidence for the Lillian 7A allele alone and 14.0% bunt incidence for the Lillian 3D allele alone. The interactions between additive × additive (epistatic) × environments were not significant in any of the 3 years.

**Table 2:** Additive × additive (AA) effects of QTL detected by two-locus interaction analysis for percent common bunt incidence in the Vesper/Lillian population evaluated near Swift Current, Canada, in 2014, 2015 and 2016

QTL 1	Interval 1	Position, cM	QTL 2	Interval 2	Position, cM	AA	SE	*p* value
Swift Current 2015								
3D	*RAC875_c3956_659*–*BobWhite_c18256_158*	144.2–145.9	7A	*Excalibur_c30730_1503*–*RFL_Contig5701_169*	207.0–211.8	1.13	0.48	0.02
Over 3 environments, Swift Current 2014–2016								
3D	*BS00065422_51*–*Excalibur_c11301_305*	145.9–146.6	7A	*wsnp_Ra_c19741_28965647*–*RAC875_rep_c119304_226*	204.5–207.0	1.09	0.33	0.001

*SE* the standard error of estimated or predicted QTL effect

^a^QTL 1 and QTL 2 are a pair of interacting QTL, 3D and 7A in this case

^b^AA designates main effect additive × additive interaction or the epistatic effect. The interactions between additive main effects and environments AA × E1, AA × E2 and AA × E3 were not significant, whereby E1, E2 and E3, respectively, represent environment 1 for the Swift Current 2014, environment 2 for the Swift Current 2015 and environment 3 for the Swift Current 2016

With five common bunt QTL, 32 unique genotypic groups exist. Table 3 is arranged by decreasing order of the 3-year mean of common bunt incidence for the 32 genotypic groups. The allelic configuration of each of the five QTL is indicated by “+” for the allele associated with reduced disease and “−” for the allele associated with more disease based on QTL analysis. The number of resistance loci per group, the number of lines per genotypic resistance group (ranging from 3 to 13) and the mean of the bunt incidence for the lines within the group in each year and across years are also indicated. Significant differences in disease incidence were observed between some genotypic groups for disease incidence using means across the 3 test years (Supplementary Table 3). Five of the six groups of lines with four or five resistance loci had significantly lower common bunt incidence when compared with five of the six groups of lines that had one or no resistance locus. The sixth groups of each Group 6 (+/+/+/−/+) and Group 27 (−/−/−/−/+) ranked beside each other medially. In some of the groups, there are lines with very different phenotypes. For example, in Group 1 with five resistance loci, line AD065 had incidence ratings of 3% in 2014, 3% in 2015 and 10% in 2016, while line AZ041 had incidence ratings of 10%, 20% and 35% for the 3 years. Group 17 (−/−/+/+/−) is split with three lines having fairly low ratings and three lines fairly high ratings. Thirteen lines are not included in Table 3 due to marker–trait recombination; for example, line AN086 carried the Vesper allele at marker *CAP8_rep_c4852_130* and the Lillian allele at *TA003720*-*0955* associated with the 5A QTL. Of the four lines that consistently had lower bunt incidence ratings than Lillian over the 3 test years, AJ091, AC037 and AH055 were categorized into Group 2 (−/+/+/+/+) while line AN089 into Group 3 (+/−/+/+/+). Among four lines that were nonsignificantly different from Biggar, line AC066 was categorized into Group 29 (−/−/+/−/−), AC005 and AN021 into Group 31 (+/−/−/−/−) and AC085 into Group 32 (−/−/−/−/−).

**Table 3:** Summary of the aggregate effects of resistance loci on bunt incidence (%) in doubled haploid lines grouped by the resistance QTL they carry and evaluated at a nursery near Swift Current from 2014 to 2016

Line group	QTL combinations 1D/2A/3D/5A/7^a^	No. of resistant loci	No. of lines in a group	Incidence 2014 (%)	Incidence 2015 (%)	Incidence 2016 (%)	Three-year mean (%)
2	−/+/+/+/+	4	12	6.7	7.5	7.3	7.2
3	+/−/+/+/+	4	4	9.3	4.8	11.3	8.4
1	+/+/+/+/+	5	8	7.5	8.6	14.4	10.2
8	−/+/−/+/+	3	8	11.3	9.8	9.8	10.3
9	+/−/−/+/+	3	7	9.3	9.0	13.6	10.6
4	+/+/−/+/+	4	6	9.2	9.5	13.3	10.7
7	+/+/+/−/−	3	11	9.0	11.8	11.8	10.8
5	+/+/+/+/−	4	12	10.6	9.4	15.4	11.8
17	−/−/+/+/−	2	6	10.2	10.5	15.8	12.2
10	−/+/+/+/−	3	11	16.1	10.5	10.5	12.4
11	+/+/−/−/+	3	10	13.8	12.3	12.3	12.8
12	−/−/+/+/+	3	9	18.9	8.3	11.1	12.8
13	+/−/+/−/+	3	13	12.0	12.2	17.9	14.0
14	+/−/+/+/−	3	11	12.8	11.8	18.2	14.3
18	+/−/−/+/−	2	7	12.4	15.0	15.7	14.4
27	−/−/−/−/+	1	10	16.0	14.8	13.5	14.8
6	+/+/+/−/+	4	3	18.3	16.7	11.7	15.6
19	−/+/−/−/+	2	5	21.0	14.0	14.0	16.3
20	−/+/−/+/−	2	11	18.1	14.5	18.9	17.2
15	+/+/−/+/−	3	12	9.8	18.9	26.3	18.3
21	+/−/+/−/−	2	9	14.4	15.6	25.0	18.3
22	+/−/−/−/+	2	8	10.8	19.1	25.0	18.3
16	−/+/+/−/+	3	7	18.9	17.4	19.3	18.5
25	−/−/+/−/+	2	5	25.0	10.2	20.6	18.6
28	−/−/−/+/−	1	5	14.0	19.0	24.0	19.0
26	−/+/+/−/−	2	7	18.6	20.7	21.4	20.2
23	−/−/−/+/+	2	4	17.5	15.8	30.0	21.1
29	−/−/+/−/−	1	9	19.3	19.4	24.4	21.1
24	+/+/−/−/−	2	5	17.4	27.4	19.0	21.3
30	−/+/−/−/−	1	8	22.5	20.0	23.8	22.1
31	+/−/−/−/−	1	9	21.7	18.7	30.6	23.6
32	−/−/−/−/−	0	9	19.0	24.4	28.9	24.1

The QTL combinations were sorted by 3-year mean bunt incidence from most resistant to most susceptible

^a^The “+” sign designates the resistance allele, while “−” sign represents susceptible allele

## Discussion

The highest disease scores of the lines and parents observed in 2016 relative to 2014 and 2015 could be attributed to the high precipitation received in 2016 favoring disease development compared to the other 2 years. The skewed continuous distribution toward resistance of the Vesper/Lillian lines (Fig. 1b) is indicative of multiple resistance genes with incomplete resistance and cumulative effects. Four of the 280 DH lines consistently expressed better resistance than the resistant parent Lillian in each of the 3 years. These lines carried all the Lillian QTL plus one of the QTL from Vesper. The identification of five minor-effect QTL in our study is consistent with the skewed phenotypic distribution. Furthermore, the moderate susceptibility of Vesper and the progeny lines which had consistently lower ratings than Lillian suggests both parents possess unique, but incomplete resistance genes, consistent with the QTL discovered. The observed QTL × environment interaction is consistent with the nature of quantitative expression (Jansen et al. 1995; Young 1996).

The *QCbt.spa*-*5A* locus is likely located on the long arm of chromosome 5A as the closely associated markers *CAP8_rep_c4852_130* and *TA003720*-*0955* map on the long arm of the chromosome in the wheat consensus map (Wang et al. 2014). We are not aware of other QTL reported for common bunt resistance on chromosome 5A; consequently, *QCbt.spa*-*5A* contributed by Lillian is likely novel. The explained variation in the bunt incidence by *QCbt.spa*-*5A* of approximately 7% was not high, but the significant expression of the locus in two out of three environments (2014 and 2015) and a reduction in bunt incidence, although not significant, in 2016 indicated the QTL is reasonably stable.

The second relatively consistent QTL *QCbt.spa*-*7A* displays a slightly larger effect on the phenotype than *QCbt.spa*-*5A*. Unlike *QCbt.spa*-*5A,* QTL with a similar effect as *QCbt.spa*-*7A* were previously reported on chromosome 7A (Dumalasová et al. 2012; Fofana et al. 2008). Fofana et al. (2008) identified a 7A bunt QTL in a Canadian wheat variety AC Domain, whereas Dumalasová et al. (2012) identified the QTL in a European winter variety Trintella. The LOD values and explained phenotypic variation of the 7A locus in Lillian, AC Domain and Trintella were also similarly modest. Dumalasová and Bartos (2012) suggested that even though the origin of varieties AC Domain and Trintella is remote, the bunt resistance could be the same based on European sources of common bunt resistance used in the USA and Canada. The two Canadian lines AC Domain and Lillian have genetic similarity with the coefficient of parentage approaching 12% [Crop Information Engine and Research Assistant (CIERA)]. Interestingly, microsatellite markers *Xgwm63* and *Xwmc633* that detected the QTL in AC Domain (Fofana et al. 2008) were monomorphic in the Vesper/Lillian population. Further work would be needed to determine whether the 7A QTL from the three sources are the same or not.

The QTL identified from Lillian and Vesper contributed approximately 35.4% of the total phenotypic variation in bunt incidence, indicating other unaccounted variation. A couple of reasons exist why the genotypic resistance group of nine lines Group 32 (−/−/−/−/−) which did not carry resistance alleles at any of the five QTL had a much lower bunt incidence (24.1%) than the average of Biggar (51.4%). One possibility is below threshold QTL were functioning but not statistically significant and secondly partial bunt resistance factors may be in common between Lillian and Vesper. The minor resistance loci identified in single years (1D in 2014, 2A in 2016 and 3D in 2015) indicate that genotype by environment interactions occurred. Interestingly, these minor QTL displayed similar LOD ratios and explained as high a level of phenotypic variation as the more consistent loci on chromosomes 5A and 7A. Steffan et al. (2017) summarizes chromosome locations of common bunt resistance genes and QTL. As with the 5A chromosome mentioned earlier, 3D, 1D and 2A were not listed as chromosomes known to be associated with common bunt resistance. Investigation of bunt resistance in contemporary varieties like Lillian is valuable for the opportunity to pyramid such genes again in newer varieties. Although multigenic, the level of resistance in Lillian is comparable to AC Cadillac which is known to have a major gene on chromosome 6D, likely *Bt10* derived from BW553 (DePauw et al. 1998; Singh et al. 2016). The improvement in bunt resistance of Lillian over previous varieties such as Neepawa indicates the success over the last few decades of persistence in resistance breeding in stacking minor-effect genes. Neepawa, related to Lillian with a 49.3% coefficient of parentage (CIERA), has been used as one of the sources of bunt resistance for many years in Canada (McCartney et al. 2013) and may be the basis of a portion of the resistance in varieties such as Lillian or even Vesper. Unfortunately, Neepawa’s resistance genes have not yet been characterized.

Generally, line groups having four or five resistance loci had significantly lower bunt incidence than those with no or one resistance locus. There were exceptions which could be attributed to small sample size or marker–trait recombination. The small sample size is exemplified by line Group 6 that had only three population lines and ranked much lower than expected for a group having four resistance alleles. Further testing would be required to determine whether the ranking of Group 6 is due to random variation associated with small sample size. Marker–trait recombination was evident in some lines of the population. Markers could be in a loose linkage with the resistance alleles, allowing marker–trait recombination that could lead to misclassification of resistant and susceptible alleles. For example, lines with the concurrent presence of the resistant allele at all five QTL did not display the lowest disease incidence. This can be explained by considering the phenotype of individual lines whereby one line of the group had a much higher bunt phenotype over all years affecting the group’s mean and ranking. The aberrant line could possess a susceptible allele or alleles as a consequence of double crossovers.

An additive effect is the reduced incidence of bunt because of the contribution of alleles at more than one locus (Singh et al. 2016). The interaction between 3D and 7A is positive, as presented in Fig. 3 and Table 2. The percent bunt incidence of the respective groups of DH lines carrying individual resistance alleles appeared higher than a simple additive effect when the 3D resistance allele was in harmony with the 7A resistance allele indicating a positive epistatic response. Genotype by environment interaction is indicated as the epistatic response was not significant in all years. Identification of significant positive (enhanced resistance) or negative (susceptible) QTL interactions is important in breeding for disease resistance to allow the deployment of allele combinations with positive synergistic effects (Singh et al. 2016, 2013).

The detection of 3D, 5A and 7A QTL with both MapQTL and QTL-Network indicates consistency in analysis between two different softwares which generates more confidence in the analysis. The manifestation of additive × additive epistatic interactions between the unstable 3D and relatively stable 7A loci in the present study indicates the importance of minor QTL in boosting resistance when combined with other QTL. The modest LOD score and low-explained phenotypic variance of 5% by the *QCbt.spa*-*3D* alone would suggest that the locus is less valuable for resistance breeding compared with the 5A and 7A loci, but this would miss the small, but positive synergistic contribution obtained through epistasis. Even though the population studied was quite large, at 280 lines, revealing five minor bunt resistance genes segregating, a larger population would assist in further elucidating the effect of additional minor loci. Although not significantly different, we identified lines that had lower disease ratings than Lillian across 3 experimental years. The genotyping showed transgressive segregation, with all four lines possessing the haplotype of the parents contributing resistant alleles at four loci compared to three resistant loci in Lillian. Sampling more environments would be useful to understand the consistency of expression of the loci and perhaps would reveal additional loci.

In conclusion, the present research revealed novel but minor-effect common bunt resistance loci that segregated from both parents of a cross of Canadian adapted varieties Lillian and Vesper. Our results suggested certain combinations of the genes are more effective. Two of the QTL located on chromosomes 3D and 7A showed at least an additive × additive epistatic effect. The results indicated resistance as good or even better than Lillian is possible through pyramiding certain combinations of the genes. Environment appeared to play a role in the expression of the gene combinations, but more environmental sampling is required to understand the best long-term gene combinations. Based on their consistency over environments, the two QTL that mapped to chromosomes 5A and 7A would be the best initial targets for marker-assisted selection as a base on which to build other loci through selection in a bunt-inoculated nursery. Lines identified through this study having both Lillian and Vesper resistance alleles could be used as future parents in further improvement in bunt resistance and variety development.
